# The use of bioinformatics methods to identify the effects of SARS-CoV-2 and influenza viruses on the regulation of gene expression in patients

**DOI:** 10.3389/fimmu.2023.1098688

**Published:** 2023-02-22

**Authors:** Zhongyi Sun, Li Ke, Qiuyue Zhao, Jiachen Qu, Yanan Hu, Han Gao, Zhiyong Peng

**Affiliations:** ^1^ Department of Critical Care Medicine, Zhongnan Hospital of Wuhan University, Wuhan, Hubei, China; ^2^ Clinical Research Center of Hubei Critical Care Medicine, Wuhan, Hubei, China; ^3^ Department of Respiratory and Critical Care Medicine, Zhongnan Hospital of Wuhan University, Wuhan, Hubei, China

**Keywords:** COVID-19, ssGSEA, influenza, protein-protein interaction, immunity

## Abstract

**Background:**

SARS-CoV-2 infection is a respiratory infectious disease similar to influenza virus infection. Numerous studies have reported similarities and differences in the clinical manifestations, laboratory tests, and mortality between these two infections. However, the genetic effects of coronavirus and influenza viruses on the host that lead to these characteristics have rarely been reported.

**Methods:**

COVID-19 (GSE157103) and influenza (GSE111368, GSE101702) datasets were downloaded from the Gene Expression Ominbus (GEO) database. Differential gene, gene set enrichment, protein-protein interaction (PPI) network, gene regulatory network, and immune cell infiltration analyses were performed to identify the critical impact of COVID-19 and influenza viruses on the regulation of host gene expression.

**Results:**

The number of differentially expressed genes in the COVID-19 patients was significantly higher than in the influenza patients. 22 common differentially expressed genes (DEGs) were identified between the COVID-19 and influenza datasets. The effects of the viruses on the regulation of host gene expression were determined using gene set enrichment and PPI network analyses. Five HUB genes were finally identified: IFI27, OASL, RSAD2, IFI6, and IFI44L.

**Conclusion:**

We identified five HUB genes between COVID-19 and influenza virus infection, which might be helpful in the diagnosis and treatment of COVID-19 and influenza. This knowledge may also guide future mechanistic studies that aim to identify pathogen-specific interventions.

## 1 Introduction

The severe acute respiratory syndrome coronavirus 2 (SARS-CoV-2) outbreak in late 2019 led to a global pandemic (1, 2). According to the World Health Organization (WHO) (https://covid19.who.int/), in August 2022, there were 594,367,247 confirmed COVID-19 cases and 6,451,016 deaths worldwide. At present, the complete impact of the COVID-19 outbreak is uncertain. Recently, SARS-CoV-2 variants Alpha (B.1.1.7), Delta (B.1.617.2), and Omicron (B.1.1.529) have also been identified one after another, becoming the main circulating strains in some countries. This has caused heavy human and economic losses worldwide (3).In addition, the beginning of the flu season can seriously affect human health. As a common respiratory pathogen, the flu causes seasonal epidemics and severe sporadic epidemics worldwide (4). The combination of the prevalence of the influenza virus during the influenza season and the current pandemic poses additional challenges and greater threats to public health.

Currently, many studies have compared COVID-19 and influenza patients, including the method and mode of transmission, clinical features, associated immune response characteristics, clinical symptoms, laboratory findings, radiological signs, morbidity, and mortality (5–8). As a respiratory infectious disease, patients with COVID-19 and influenza experience the same or similar symptoms, including fever, cough, pneumonia, acute respiratory distress syndrome, an imbalanced immune response, excessive inflammatory response, T-cell depletion and failure, and immune escape mechanisms (6, 8, 9). However, influenza virus infection results from a direct viral infection of respiratory epithelial cells and a respiratory inflammatory process caused by innate and adaptive immune responses, the main purpose of which is to control the spread of the transmitted virus (7). Inflammatory mediators can spread throughout the body, causing systemic inflammatory response syndrome (SIRS) and leading to multiple organ failure. These consequences are often downstream of lung damage and severe respiratory distress. Other non-pulmonary disease mechanisms associated with influenza are also thought to be associated with general inflammatory features (7, 10). Severe COVID-19 results in damage to the alveolar capillary barrier caused by severe acute respiratory syndrome coronavirus 2 (SARS-CoV-2) infection and extravasation of protein-rich edematous fluid into the air cavity, resulting in acute respiratory distress syndrome (ARDS) (11, 12). ARDS is a systemic inflammatory disease that is not confined to pulmonary processes. In this case, the cytokine storm induced by COVID-19 leads to the worsening and even death from COVID-19, not only because of lung damage but also because of extrapulmonary multi-organ failure (7, 13). The basic reproductive number R zero (R0) of COVID-19 (1.5-5.7) is more significant than that of influenza (0.9-2.1) (14). At the same time, the transmission rate of SARS-CoV-2 is higher than that of seasonal influenza, but the mortality rate of the latter is much lower than that of COVID-19 (6, 8). Chemosensory dysfunction, rashes, and reproductive system damage are more common in people infected with COVID-19 than in those with influenza (6, 15). Numerous reports illustrate the similarities and differences between COVID-19 and influenza. However, the genetic effects of the coronavirus and influenza viruses on the host that lead to these characteristics have rarely been reported. Comprehensive assessment of host regulation of gene expression in both diseases can be used to identify the populations at high risk, enhance our focus on specific preventive measures for these populations, and help define future needs for healthcare facilities.

To better understand the effects of coronavirus and influenza virus infection on the changes of host mRNA levels and biological processes. And explore the interconnections between different influences as much as possible. We obtained sequencing data from the GEO database for influenza (GSE111368, GSE101702) and COVID-19 (GSE157103) (16). Explored the differential genes in COVID-19 and influenza patients using bioinformatics methods, identified hub genes, and explored pathway biological processes and pathways that influence each of these diseases to gain a more comprehensive understanding of the host response to SARS-CoV-2 and influenza viruses. The results of this study will help us understand the association between gene expression and clinical manifestations, which will improve our ability to develop effective treatment methods for infected patients.

## 2 Materials and methods

### 2.1 Data collection

The expression datasets of COVID-19 and influenza patients were retrieved from the GEO database (https://www.ncbi.nlm.nih.gov/). GSE157103 contained data on 100 patients who tested positive for COVID-19 and 26 controls, while GPL24676 was used to detect the mRNA expression profiles. GSE111368 included data from 199 patients with H1N1 influenza virus infection, 30 patients with other influenza virus infection, and 130 controls, while GPL10558 was used to detect the mRNA expression profile. GSE101702 contained data on 57 healthy controls and 102 influenza patients, while GPL21185 was used to detect the mRNA expression profile. The information of the datasets was listed in **Table 1**.

**Table 1:** Detailed information of selected datasets.

Disease name	COVID-19	Influenza	Influenza
GEO accession	GSE157103	GSE111368	GSE101702
GEO platform	GPL24676	GPL10558	GPL21185
Tissue (Homo sapiens)	Peripheral Blood	Peripheral Blood	Peripheral Blood
Experiment Type	RNA-Seq	Array	Array
Number of samples	100 patients and 26 controls	199 patients had H1N1 influenza virus infection, 30 patients had other influenza virus infection and 130 controls	102 patients and 57 controls
Country	USA	United Kingdom	Germany
Description	Patients tested positive for COVID-19	The majority had H1N1 influenza virus infection	World Health Organization definition of influenza-like illness (fever of 38 °C or higher, cough and illness onset within the last ten days). Eligible patients were assessed by an admitting physician for likelihood of influenza infection.

### 2.2 Analysis of differentially expressed genes

Raw sequencing data were first retrieved from the GEO database. After normalization, the raw sequencing data were log_2_-transformed, and the resulting array was directly analyzed using the R package “limma” (17). An adjusted-*p* value of < 0.05 and log_2_[fold change (FC)] > 1 were considered to indicate a statistically significant result and were used in the subsequent analysis. The heatmap of the top 15 genes with high and low expression differences were drawn using the R package “pheatmap.” The differential volcano map was drawn using the R package “ggplot2”.

### 2.3 Enrichment analysis

Next, we performed GO and KEGG enrichment analyses of the differential genes in three datasets using the R packages “clusterProfiler” and “Enrichplot.” We explored the associations and distinctions between the diseases by comparing the enrichment of the differential genes in each dataset. A *p* < 0.05 and adjusted-*p* < 0.05 indicated significantly enriched functions and pathways.

### 2.4 Hub gene extraction

To evaluate the common DEGs among the three diseases, the R packages “VennDiagram” and “UpSetR” were used to draw the Venn and UpSet diagrams of the intersection of the genes. The interaction between intersecting genes was analyzed using the STRING database (18). Cytoscape software was used to compute the network degree and draw the interaction diagram (19). The MCC algorithm of the cytoHubba plugin in Cytoscape was used to calculate the top 5 hub genes and draw the relevant images (20). Additionally, we verified the identity of the hub genes using differential and ROC analyses (21).

### 2.5 Association between the analysis of the gene regulatory network and gene-diseases

Transcription factors (TFs) and miRNAs are the two main types of trans-acting factors. They are essential regulators of gene expression that play an important role in cell differentiation, animal growth, and development. NetworkAnalyst is a web tool that can comprehensively visualize profile data (22). We used NetworkAnalyst to explore the TFs obtained from the JASPAR (23) database and miRNA data from the TarBase and miRTarBase databases to determine the intersecting genes (24, 25). We also explored the interrelationships between the genes in our datasets and known diseases using DisGeNet in NetworkAnalyst (26).

### 2.6 Immune correlation analysis

The degree of immune cell infiltration in each sample was determined through single-sample gene set enrichment analysis (ssGSEA) using the R package, “GSVA.” The Wilcoxon rank-sum test was used to compare differences in immune cells between the disease and control groups. Spearman analysis was used to evaluate the correlation between hub genes and immune cells. A *p* < 0.05 was considered to indicate statistical significance.

### 2.7 Statistical analysis

R software (v.4.2.0) was used to perform all statistical analyses and mapping. The differentially expressed genes were analyzed using Student’s t-test, while the Spearman correlation coefficient was used to evaluate the relationship between hub genes and immune cells. The difference was considered to be statistically significant at p<0.05 (^*^), p<0.01 (^**^), and p<0.001 (^***^).

## 3 Results

### 3.1 Heat map and volcano map of differential gene expression in COVID-19 and influenza datasets

**Figure 1:**
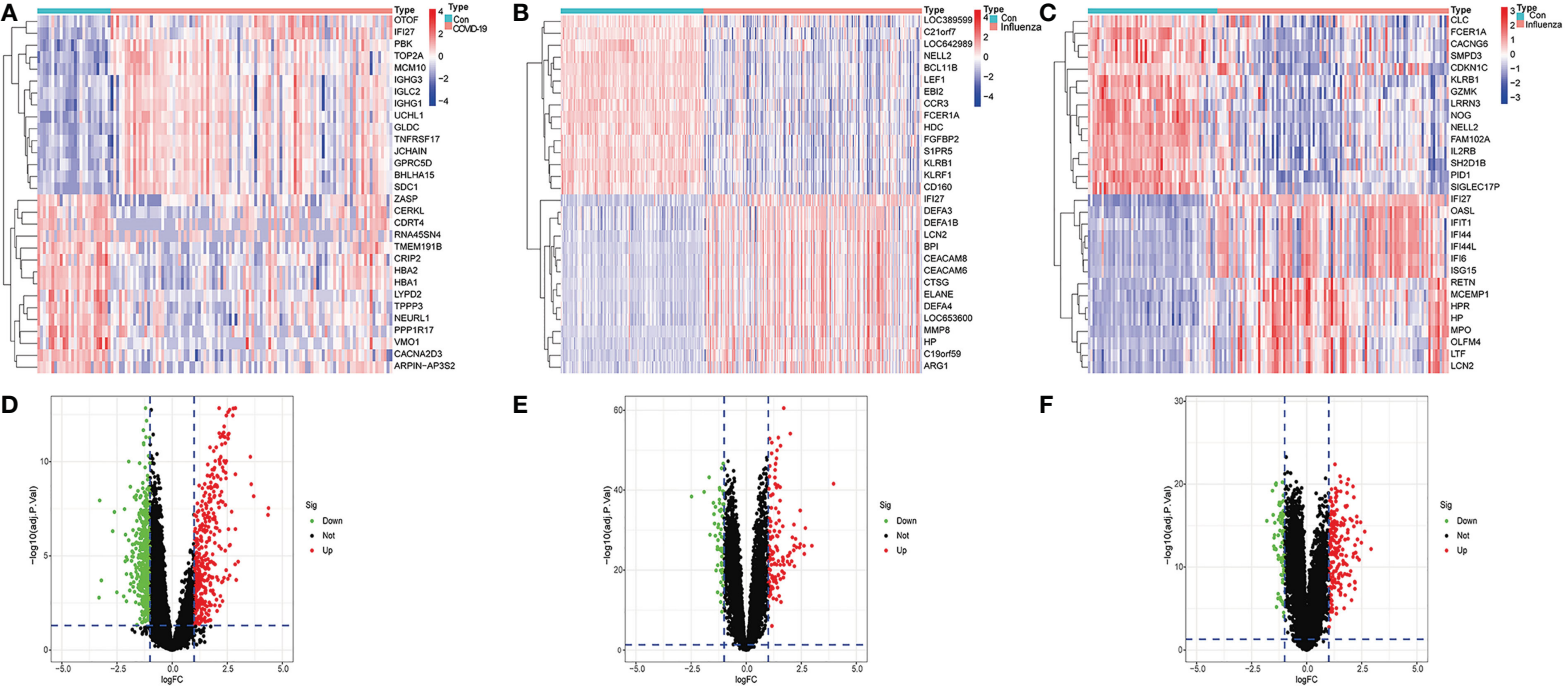
Identification of differentially expressed genes. **(A)** Heatmap of GSE157103 database, **(B)** Heatmap of GSE111368 database, **(C)** Heatmap of GSE101702 database, **(D)** Volcano of GSE157103 database, **(E)** Volcano of GSE111368 database **(F)** Volcano of GSE101702 database.

We identified 974 differentially expressed genes in the GSE157103 dataset, with 395 upregulated and 579 downregulated. The heat map of the top 15 DEGs with high and low expression differences in the GSE157103 dataset was presented in **Figure 1A**. The differential expression volcano map of all genes is shown in **Figure 1D**. We found 190 DEGs in the GSE111368 dataset, with 129 upregulated and 61 downregulated. **Figure 1B** shows the top 15 DEGs of H1N1 virus infected patients and controls in the GSE111368 dataset, while the volcano map with all DEGs is presented in **Figure 1E**. Additionally, 275 DEGs were identified in the GSE101702 dataset, with 197 upregulated and 78 downregulated. The heat map of the top 15 DEGs in the GSE171110 dataset is presented in **Figure 1C**, and the volcano map of all the DEGs is shown in **Figure 1F**. The number of differentially expressed genes in COVID-19 patients was significantly higher than that of influenza patients, which may lead to an increase in complex clinical symptoms in COVID-19 patients.

### 3.2 GO functional enrichment analysis and KEGG pathway analysis of differentially expressed genes in COVID-19 and influenza datasets

**Figure 2:**
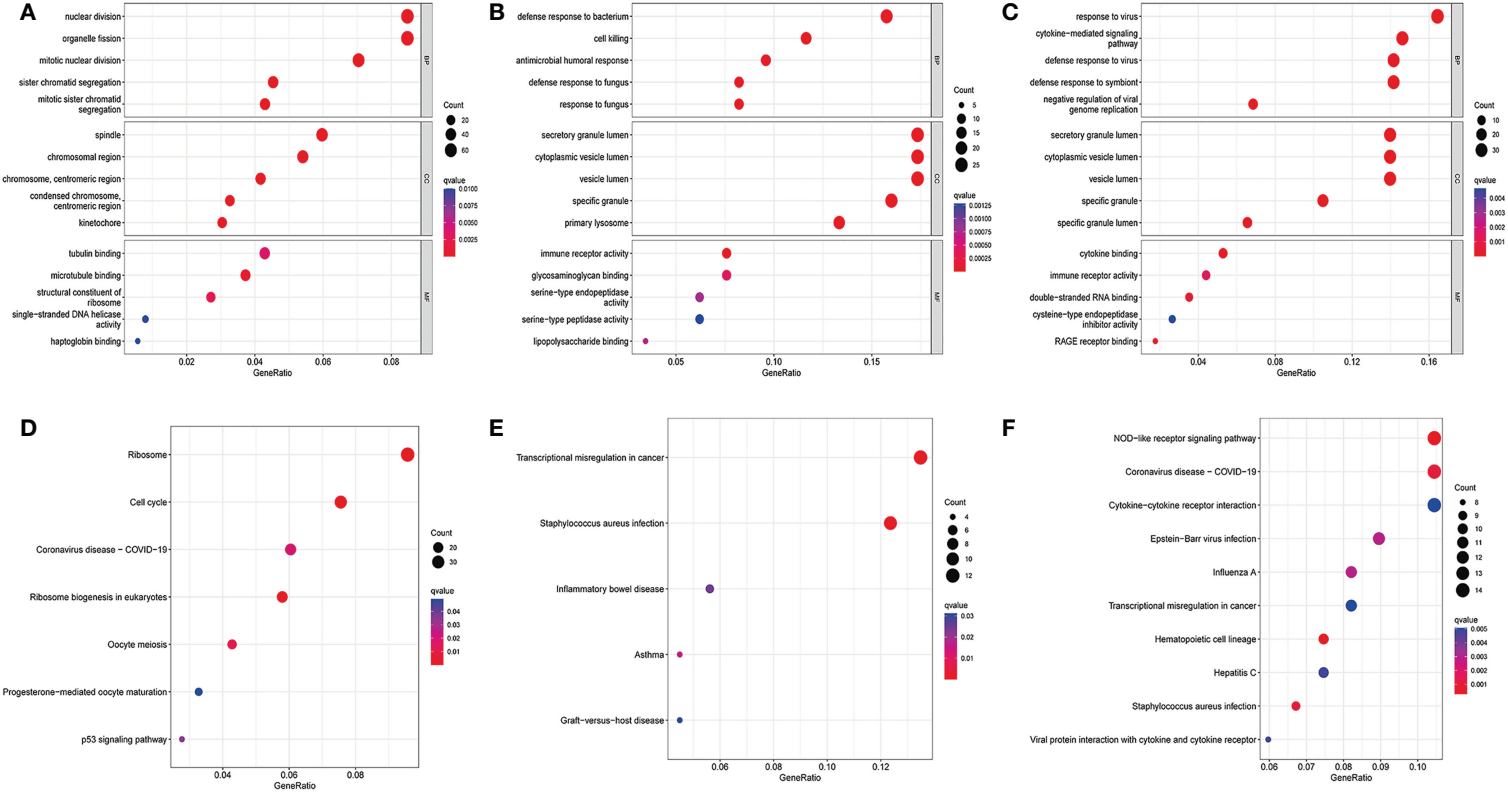
GO enrichment analysis and KEGG enrichment analysis. **(A)** Bubble for GO enrichment analysis of differentially expressed genes in the GSE157103 dataset, **(B)** Bubble for GO enrichment analysis of differentially expressed genes in the GSE111368 dataset, **(C)** Bubble for GO enrichment analysis of differentially expressed genes in the GSE101702 dataset, **(D)** Bubble for KEGG enrichment analysis of differentially expressed genes in the GSE157103 dataset, **(E)** Bubble for KEGG enrichment analysis of differentially expressed genes in the GSE111368 dataset, **(F)** Bubble for KEGG enrichment analysis of differentially expressed genes in the GSE101702 dataset.

Next, we performed the GO functional analysis of the DEGs in the COVID-19 and influenza datasets. The top 5 most significant enrichment results in cellular components (CCs), molecular functions (MFs), and biological processes (BPs) are presented in **Figure 2**. In the COVID-19 dataset, BPs were mainly enriched in nuclear division and organelle, CCs were mainly enriched in the Spindle chromosomal region, and MFs were mainly enriched in tubulin binding and microtubule binding (**Figure 2A**). This may lead to the overproduction of inflammatory cytokines in COVID-19 patients. In the GSE111368 dataset, BPs were mainly enriched in Defense response to bacterium and cell killing, CCs were mainly enriched in secretory mutlumen and cytoplasmic vesicle lumen, and MFs were mainly enriched in immune receptor activity and glycosaminoglycan binding (**Figure 2B**). In the GSE101702 dataset, BPs were mainly concentrated on response to viruses, defense, and response to viruses; CCs were mainly concentrated on secretory mutlumen and cytoplasmic vesicle lumen, while MFs were mainly concentrated on cytokine binding immune receptor activity (**Figure 2C**). GO results showed that influenza virus infection mainly caused active expression of genes related to self-defense and immune system inhibition of virus replication and inflammatory response.

The KEGG pathway analysis was also performed on the DEGs in the COVID-19 and influenza datasets. The most enriched pathways in each of the three datasets are shown in (**Figures 2D–F**). The COVID DEGs were mainly enriched in Ribosome and Cell cycle (**Figure 2D**). The GSE111368 dataset DEGs were mainly enriched in transcriptional misregulation in cancer, *Staphylococcus aureus* infection, and inflammatory bowel disease (**Figure 2E**). The GSE101702dataset DEGs were mainly enriched in the NOD-like receptor signaling pathway, coronavirus disease-COVID-19, Cytokines, and Cytokine receptor interaction (**Figure 2F**).

### 3.3 Molecular functional analysis of intersecting DEGs

**Figure 3:**
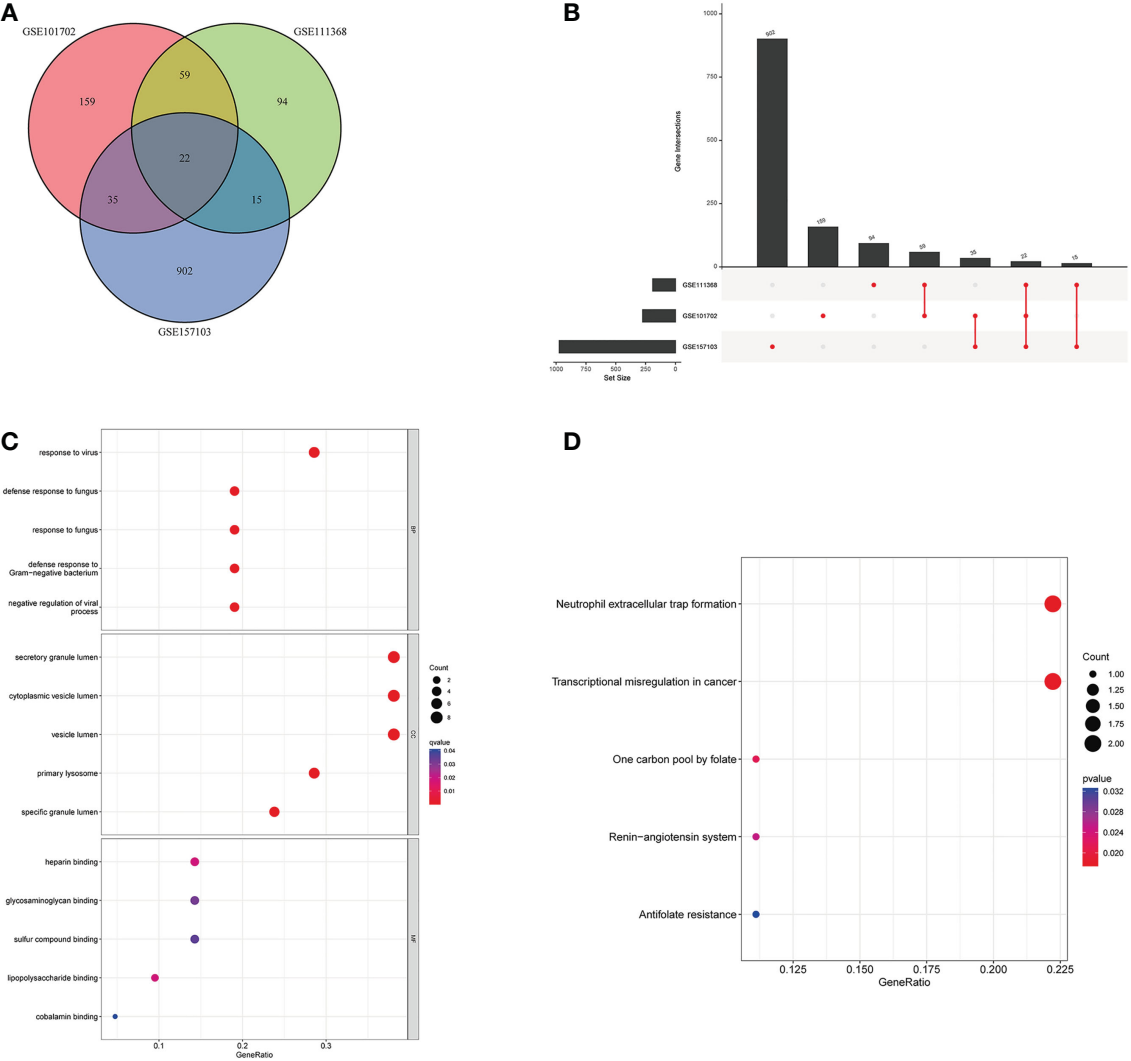
Molecular functional analysis of intersecting DEGs. **(A)** Intersectional differential gene Venn diagram, **(B)** Intersectional differential gene Up Set diagram, **(C)** Bubble for GO enrichment analysis of DEGs, **(D)** Bubble for KEGG enrichment analysis of DEGs.

To further explore the potential common pathogenic molecular mechanisms between the three diseases, we used Venn and Upset diagrams to detect 22 common DEGs among the three datasets (**Figures 3A, B**). Then, we performed GO and KEGG analyses on these genes. The common DEGs were mainly enriched for BPs in defense response to fungus, response to fungus, and defense response to Gram-negative bacterium. For CCs, the common DEGs were enriched in secretory mutlumen, cytoplasmic vesicle lumen, and vesicle lumen. For MFs, the common DEGs were mainly enriched in Heparin binding, Glycosaminoglycan binding, and sulfur compound binding (**Figure 3C**). The KEGG pathways were mainly enriched in Neutrophil extracellular trap formation, Transcriptional misregulation in cancer, and one carbon pool by folate (**Figure 3D**).

### 3.4 Differential expression analysis of the 22 genes

**Figure 4:**
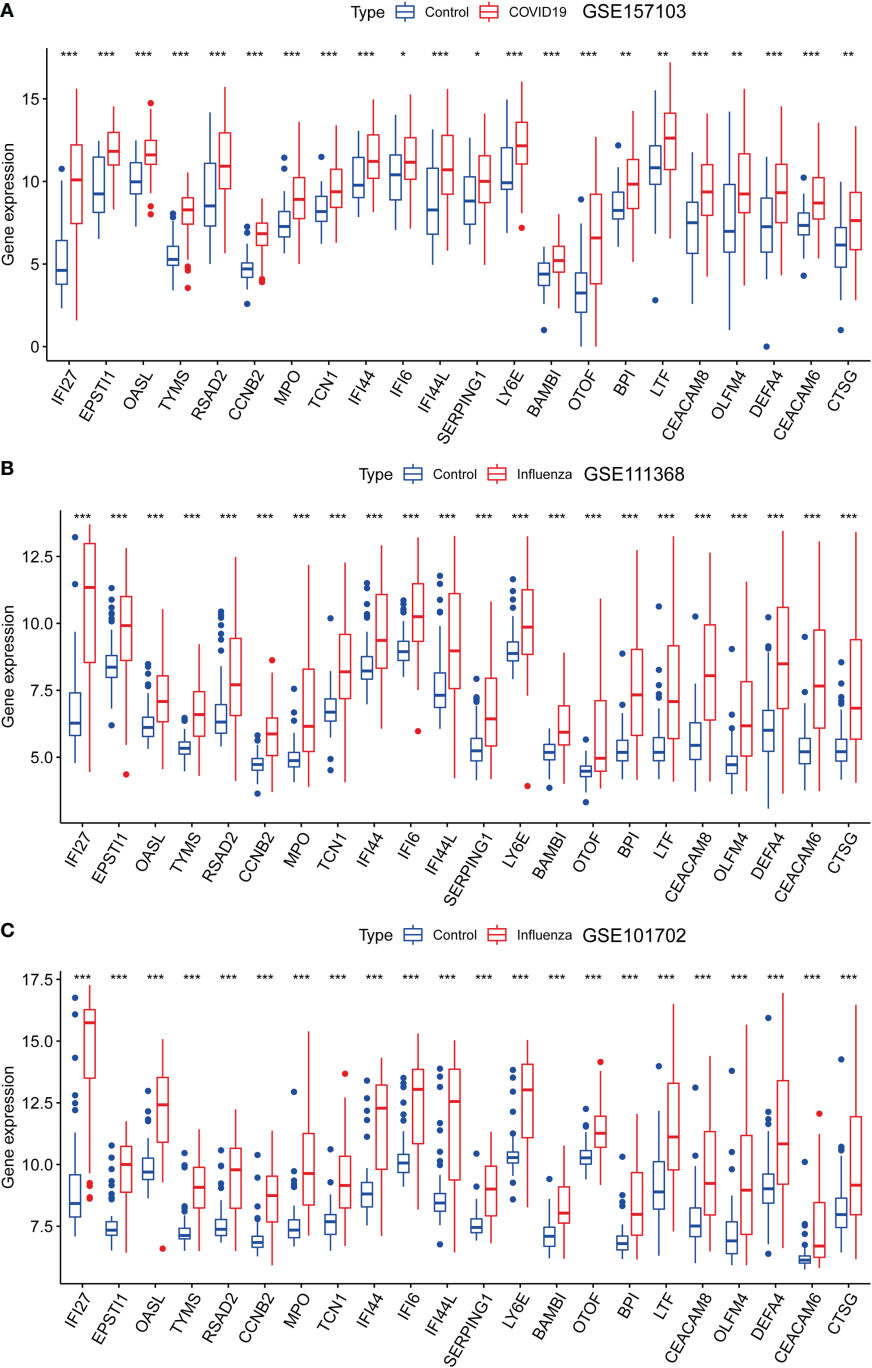
Expression analysis of the 22 DEGs in GSE157103, GSE111368, and GSE101702 datasets between types of severity and healthy control groups. **(A)** mRNA expression levels of 22 DEGs between control and COVID-19 in GSE157103 dataset, **(B)** mRNA expression levels of 22 DEGs between control and influenza in GSE111368 dataset, **(C)** mRNA expression levels of 22 DEGs between control and influenza in GSE101702 dataset (*p<0.05, **p<0.01, ***p<0.001).

We analyzed the expression levels of the 22 genes in the GSE157103, GSE111368, and GSE101702 datasets. As shown in **Figure 4**, the 22 common DEGs represent the same host responses to SARS-CoV-2 and influenza infections. All of them were significantly upregulated in the COVID-19, H1N1, and influenza groups compared to the healthy controls (P < 0.05) (**Figures 4A–C**).

### 3.5 Molecular regulation of DEGs at the transcriptional and post-transcriptional level

**Figure 5:**
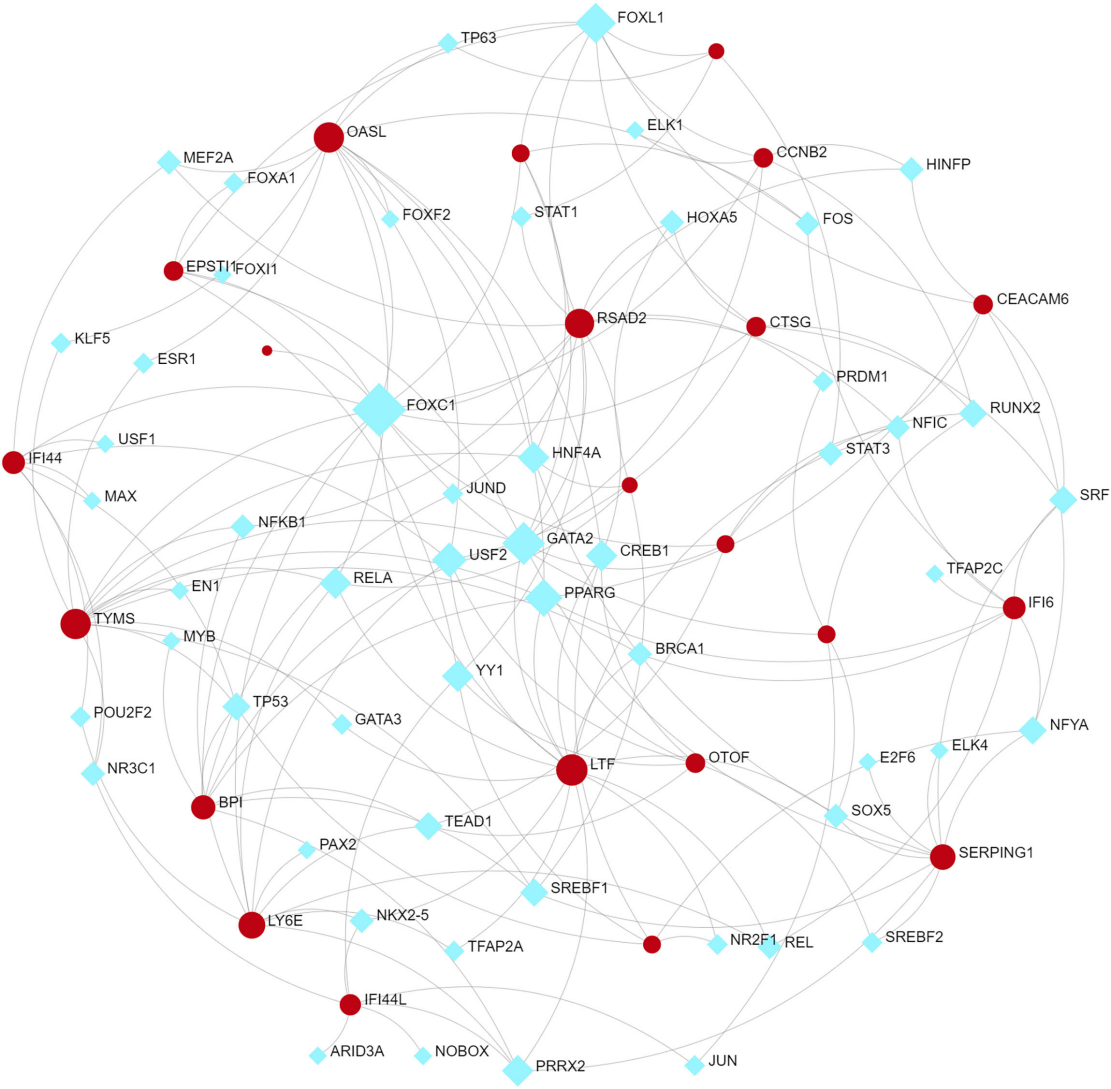
The cohesive regulatory interaction network of DEG–TFs obtained from the Network Analyst.

**Figure 6:**
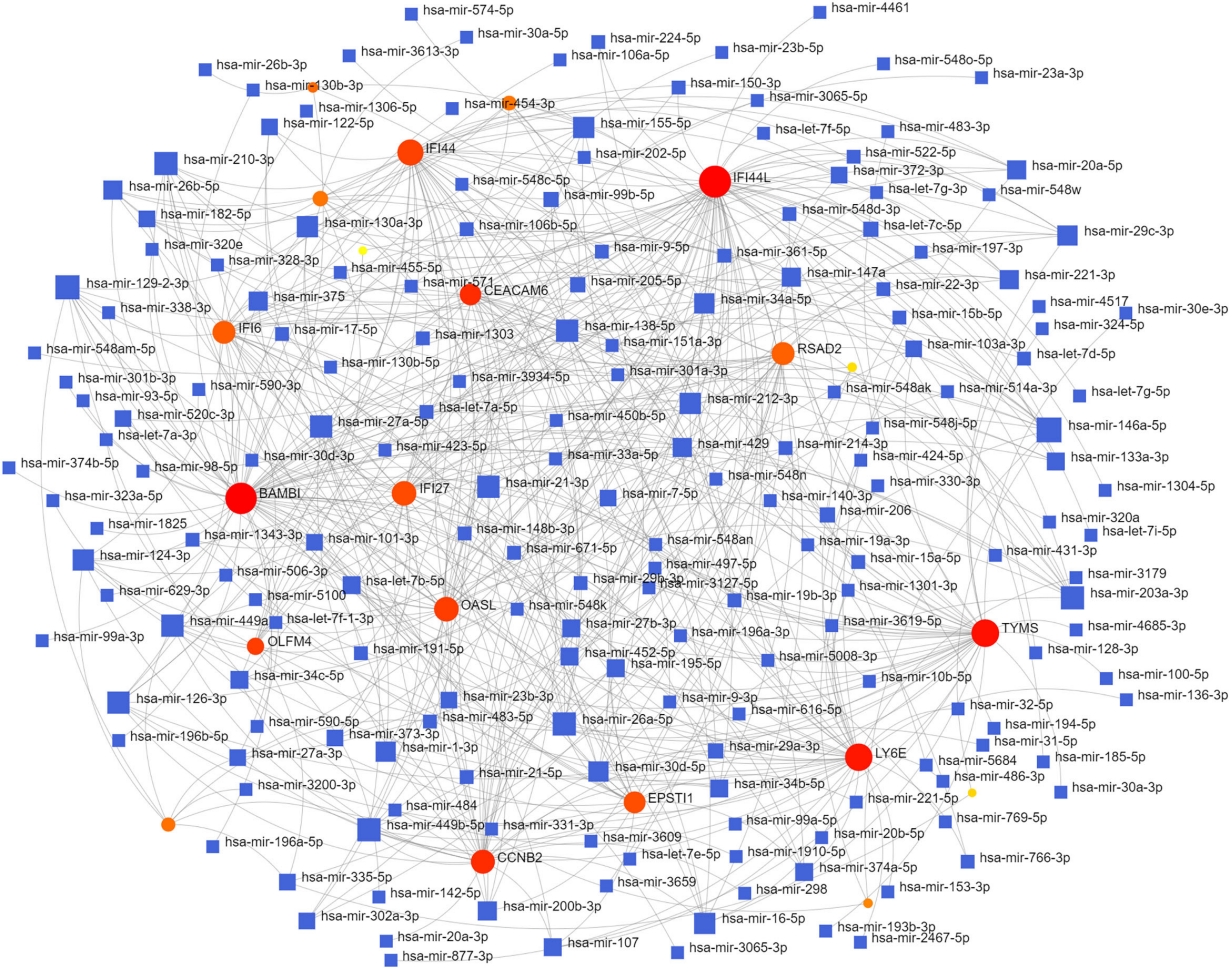
The interconnected regulatory interaction network of DEGs–miRNAs.

**Figure 7:**
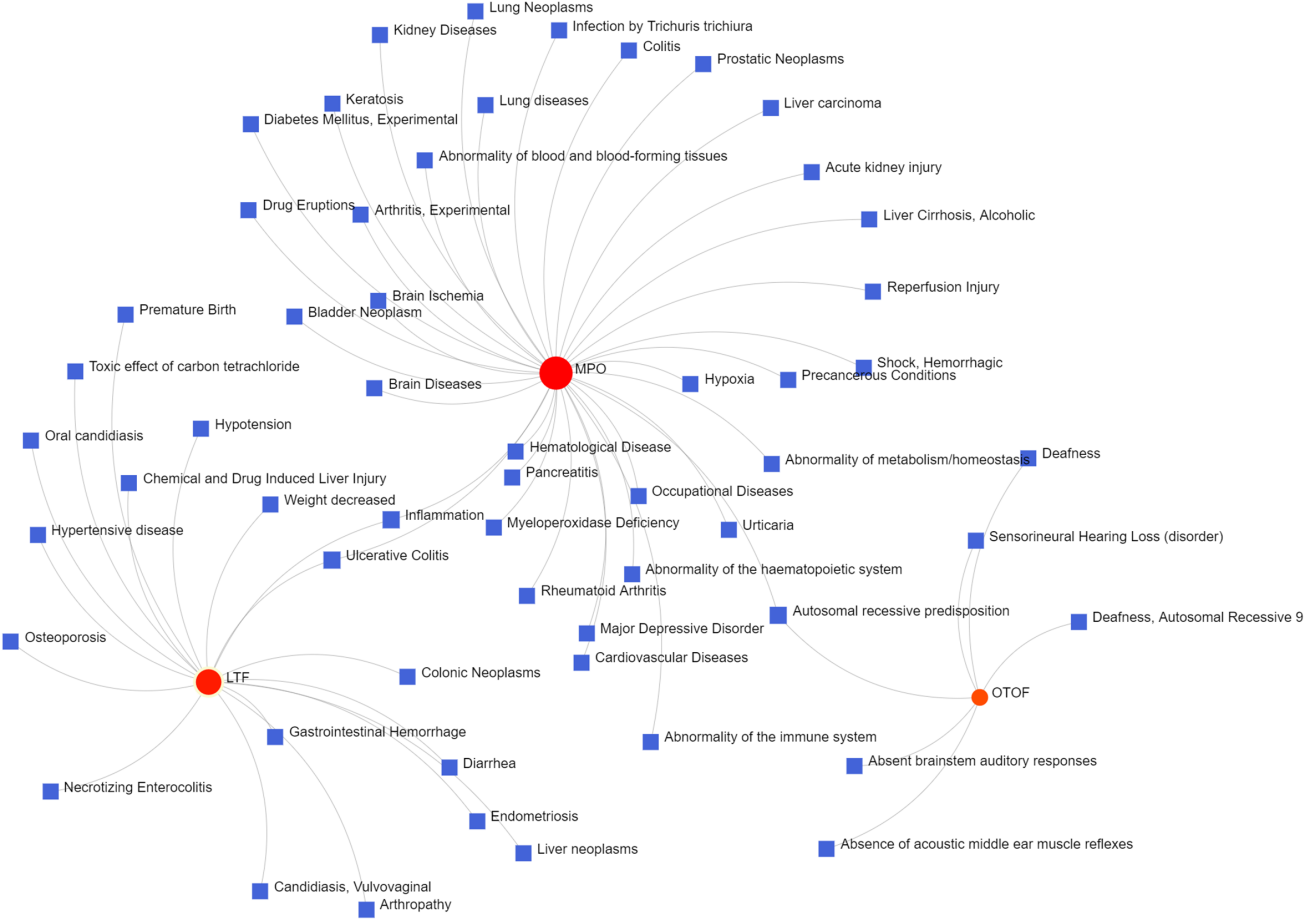
The gene-disease association network represents diseases associated with mutual DEGs.

To identify changes in the DEGs at the transcriptional and post-transcriptional level of molecular regulation, we used a network-based approach and NetworkAnalyst to decipher regulatory transcription factors and miRNAs. The interaction network analysis showed that 54 transcription factors (TFs) (**Figure 5**) and 187 post-transcriptional miRNAs (**Figure 6**) were involved in the regulation of several common DEGs, indicating a substantial level of interference between them. Different diseases may be related through the same or similar genes and deciphering the relationship between genes and diseases is a key approach to disease diagnosis and treatment. Our genetic disease association analysis found that ulcerative colitis, inflammation, autosomal recessive predisposition, and other diseases are strongly associated with the intersecting genes (**Figure 7**).

### 3.6 PPI network and hub gene

**Figure 8:**
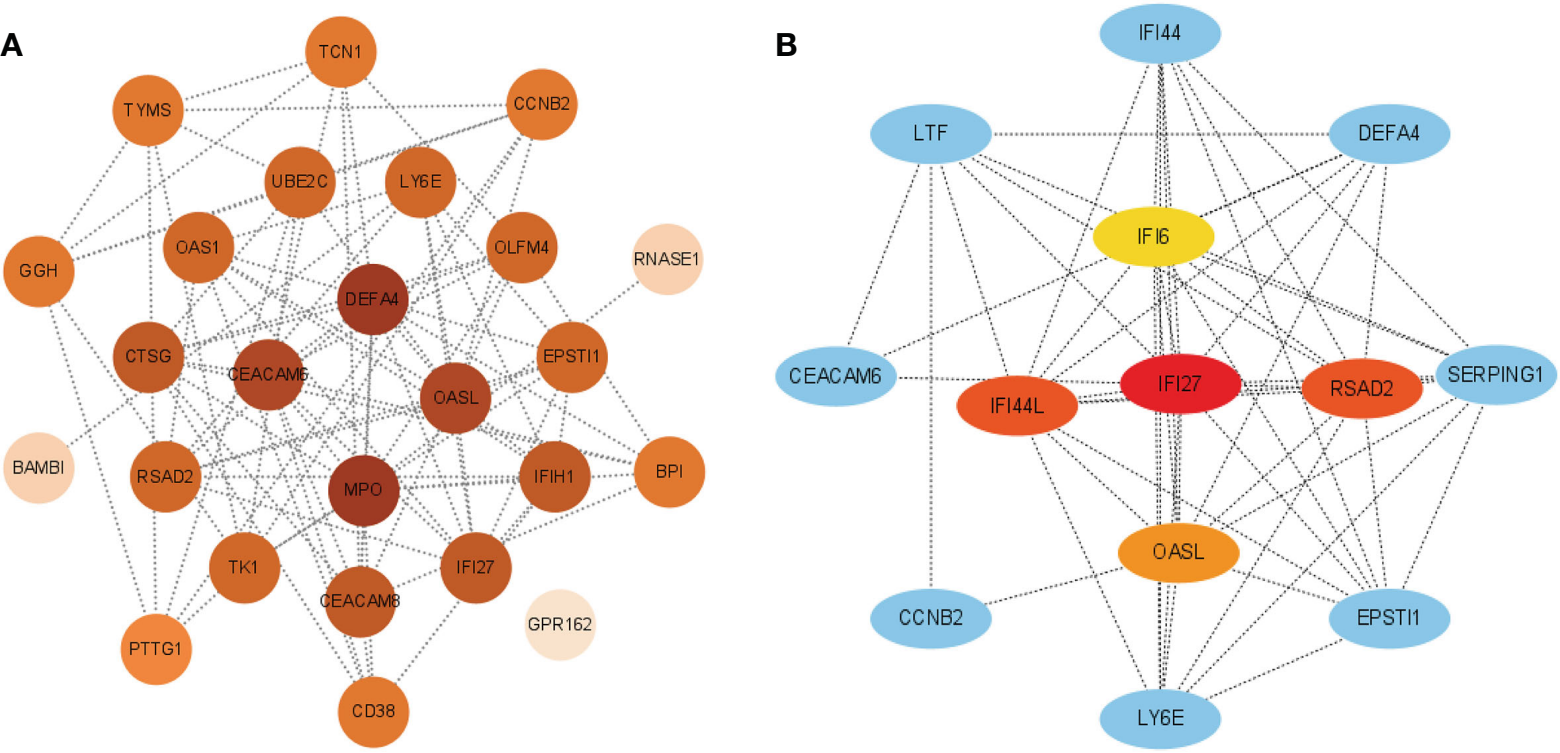
**(A)** PPI network of common DEGs among SARS-CoV-2, H1N1, and Influenza. The PPI network was generated using String and visualized in Cytoscape. **(B)** Determination of hub genes from the PPI network by using the Cytohubba plugin in Cytosacpe.

**Figure 9:**
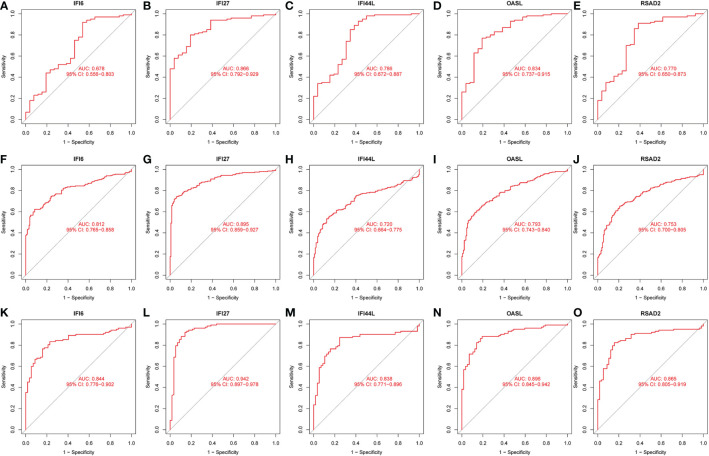
ROC analysis of HUB gene. **(A)** ROC of IFI6 in the GSE157103 dataset. **(B)** ROC of IFI27 in the GSE157103 dataset. **(C)** ROC of IFI44L in the GSE157103 dataset. **(D)** ROC of OASL in the GSE157103 dataset. **(E)** ROC of RSAD2 in the GSE157103 dataset. **(F)** ROC of IFI6 in the GSE111368 dataset. **(G)** ROC of IFI27 in the GSE111368 dataset. **(H)** ROC of IFI44L in the GSE111368 dataset. **(I)** ROC of OASL in the GSE111368 dataset. **(J)** ROC of RSAD2 in the GSE111368 dataset. **(K)** ROC of IFI6 in the GSE101702 dataset. **(L)** ROC of IFI27 in the GSE101702 dataset. **(M)** ROC of IFI44L in the GSE101702 dataset. **(N)** ROC of OASL in the GSE101702 dataset. **(O)** ROC of RSAD2 in the GSE101702 dataset.

The interactions between intersecting genes were analyzed using the STRING online database. The degree of interaction was calculated using Cytoscape software, and the relationships between the proteins are displayed based on the strength of the interaction in **Figure 8A**. The Cytohubba plugin in Cytoscape was used to identify the top five DEGs, regarded as the most influential genes based on the PPI network and MCC algorithm. IFI27, OASL, RSAD2, IFI6, and IFI44L were identified as the hub genes in **Figure 8B**. ROC analysis of hub genes was used to identify the diagnostic efficacy of hub genes in the COVID-19 dataset and influenza dataset. The ROC analysis of the hub genes is presented in **Figure 9**. The AUC of genes in the COVID-19 cohort are as follows: IFI6, 0.678; IFI27, 0.866; IFI44L, 0.786; OASL, 0.834; RSAD2, 0.770 (**Figures 9A–E**). The AUC of genes in the GSE111368 H1N1 dataset are as follows: IFI6, 0.812; IFI27, 0.895; IFI44L, 0.720; OASL, 0.793; RSAD2, 0.753 (**Figures 9F–J**). The AUC of genes in the GSE101702influenza datasets are as follows: IFI6, 0.844; IFI27, 0.942; IFI44L, 0.838; OASL, 0.896; RSAD2, 0.865 (**Figures 9K–O**). Thus, these hub genes may be viable biomarkers and be used to develop novel therapeutic strategies for these diseases. We also compared the expression of hub genes between healthy controls and different severity groups. The results showed that there was no significant difference in the expression of hub genes between patients with different severity in the COVID-19 dataset (P>0.05), while there was a significant difference in the expression of hub genes between healthy controls and influenza patients in the influenza dataset (P<0.05). (**Supplementary Figure 1**).

### 3.7 Immune cell correlation of hub genes in COVID-19 dataset and influenza dataset

**Figure 10:**
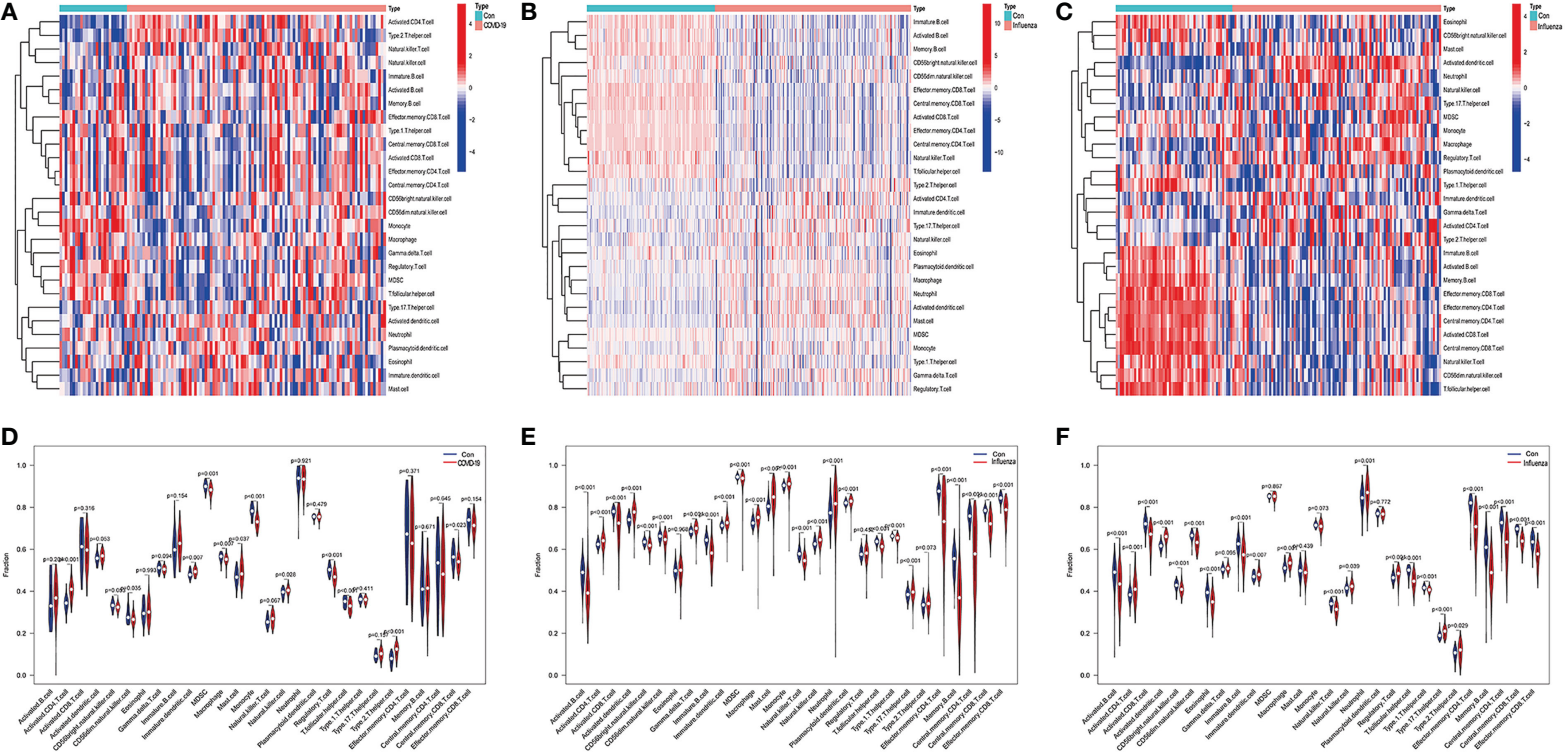
**(A)** Heatmap of infiltrated immune cells in COVID-19, **(B)** Heatmap of infiltrated immune cells in H1N1, **(C)** Heatmap of infiltrated immune cells in Influenza. **(D)** Fraction of infiltrated immune cells in COVID-19, **(E)** Fraction of infiltrated immune cells in H1N1, **(F)** Fraction of infiltrated immune cells in Influenza.

**Figure 11:**
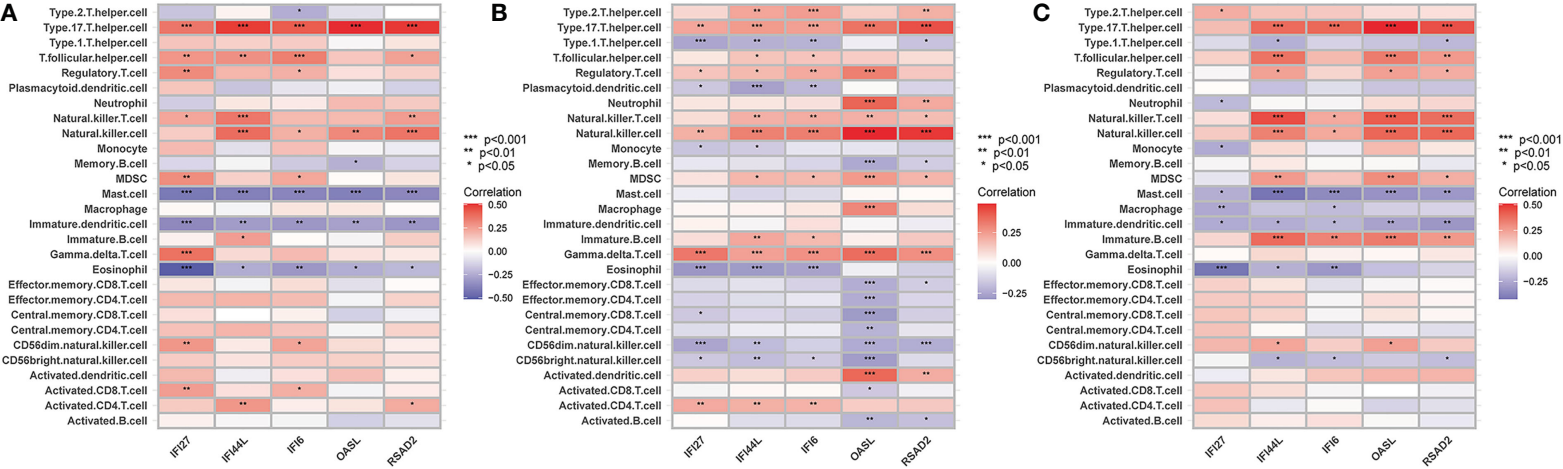
**(A)** The correlation between hub genes-immune cells and the fraction of infiltrated immune cells in COVID-19, **(B)** The correlation between hub genes-immune cells and the fraction of infiltrated immune cells in H1N1, **(C)** The correlation between hub genes-immune cells and the fraction of infiltrated immune cells in Influenza. *p < 0.05, **p < 0.01, ***p < 0.001.

The ssGSEA results are presented in **Figure 10**. The numbers of activated CD8 T cells, effector memory CD4 T cells, memory B cells, and central memory CD8 T cells were significantly reduced in influenza and COVID patients. Moreover, Activated CD4 T Immature dendritic cells, Macrophages, and Natural killer cell levels were significantly elevated in the disease groups compared with the control group. IFI27 was negatively correlated with Eosinophils. IFI44L was positively correlated with Type 17 T helper, T follicular helper, and Natural killer cells. IFI6 was positively correlated with Type 17 T helper and Natural killer cells, with OASL and RSAD2 showing a similar result (**Figures 11A–C**).

## 4 Discussion

COVID-19 and influenza are infectious respiratory diseases that can be deadly (27, 28). The pathogenicity of COVID-19 and influenza and the regulation of gene expression in the host cause them to present the same or similar clinical manifestations. Several studies have shown an association between IAV and SARS-CoV-2. However, a comparison of gene expression regulation between the two viruses on the host has rarely been reported. To comprehensively evaluate the effects of these two viruses on the regulation of host gene expression, we downloaded the sequencing data of influenza (GSE111368, GSE101702) and COVID-19 (GSE157103) from the GEO database. The analysis of the differentially expressed genes showed that the number of differentially expressed genes in COVID-19 patients was significantly higher than that of influenza patients, which may lead to an increase in complex clinical symptoms in COVID-19 patients. This is in line with the finding by Bai and Ryabkova that COVID-19 is a systemic inflammatory disease that is not confined to pulmonary processes, compared with influenza (6, 7). The GO results showed that functions of DEGs in the COVID-19 dataset were significantly enriched in the mitotic nuclear division, nuclear division, specific mutational division, and microtubule binding. This may lead to the overproduction of inflammatory cytokines in patients, which is consistent with the findings of Varvara et al. (7). The DEGs in the GSE111368 dataset were significantly enriched in defense response to fungus, defense response to bacteria, specific accumulation, and immune receptor activity. The functions of the differentially expressed genes in GSE101702 were significantly enriched in negative regulation of viral genome replication, defense response to viruses, defense response to the symbiont, and immune receptor activity. This is consistent with the study by Kalil et al. that the pathogenesis of influenza viral infection is a respiratory inflammatory process caused by a direct viral infection of respiratory epithelial cells that stimulates innate and adaptive immune responses, whose main purpose is to control the transmitted disease (29). We found a similar process in the same KEGG analysis of the COVID-19 dataset, which was mainly enriched in cell cycle and other processes. In contrast, the GSE111368 dataset was mainly enriched in *Staphylococcus aureus* infection. For transcriptional misregulation in cancer, GSE101702 was mainly enriched in the Nod-like receptor signaling pathway and *Staphylococcus aureus* infection. These results indicate that the SARS-CoV-2 and influenza viruses differ at the transcriptome level. The novel coronavirus may cause changes in the cell cycle and proliferation to resist infections by changing the level of metabolism. The differential genes of the influenza virus may play a more important role in activating immunity and immune response in the body. These results reveal differences between SARS-CoV-2 and influenza viral infection from the perspective of gene expression regulation. We identified 22 common differential genes between the COVID and influenza datasets. The GO and KEGG analyses showed that these genes were mainly involved in fungus defense response, defense response to Gram-negative bacterium, glycosaminoglycan binding, and Sulfur compound binding. This suggests that the common genes may be responsible for fever, cough, pneumonia, and other clinical manifestations in influenza and COVID patients (6, 8, 11).

Furthermore, TFs and miRNAs are two key regulatory factors at the transcriptional and post-transcriptional levels. The regulatory network formed with TFs and miRNAs as the core is effective for analyzing the complexity of biological regulation. Thus, studying the regulatory network composed of TF and miRNA might provide important clues for the occurrence and pathogenesis of diseases at the system level. In the TFs-DEG and miRNA interaction analysis, we found associations between intersecting genes, TFs, and miRNAs. TP63, FOXA1, STAT1, ELK1, FOS, and JUN are TFs in various lung injury or infection types. Some studies have shown that TP63 is involved in airway repair following injury (30), while FOXA1 plays an important role in maintaining airway epithelial barrier integrity and lung cell differentiation (31, 32). The AK2/STAT1 pathway mediated lung inflammation and cell death in a ventilator-induced lung injury model (27). The intersecting genes-miRNA analysis showed that miRNA302, miRNA126, miRNA21, miRNA486, and miRNA206 are associated with the pathogenesis of various types of lung injury. miRNA302 promotes host recovery from pneumonia caused by *Streptococcus pneumoniae (* 28), while miRNA126 attenuates LPS-induced lung injury and may be involved in the pathogenesis of asthma (33, 34). miRNA21 is a potential biomarker of chronic lung disease in preterm infants and may also be a potential biomarker of lung nodules (35, 36). Currently, little is known about the role played by miRNAs and TFs in the pathogenesis of COVID-19. Previous studies have shown that most TFs and miRNAs are significantly upregulated in patients with COVID-19 and influenza (37, 38). The upregulated miRNAs and TFs may be involved in the inflammatory storm of patients and can be used as circulating biomarkers for disease diagnosis or prognosis (39). Currently, 19 TFS have already received FDA approval to be used as drug targets for COVID-19 (40). Targeting TFs and miRNAs associated with cytokine release syndromes may provide drug candidates and targets for treating influenza and COVID-19 infections. However, further research is needed to confirm these findings.

Then, we performed a PPI network analysis. Five hub genes were identified in Cytoscape software using the MCC algorithm of cytoHubba: IFI27, IFI44L, RSAD2, OSAL, and IFI6. The ROC analysis showed that the AUC of all Hub genes was > 0.6, suggesting they may be potential biomarkers. Studies have found that IFFI44, IFI6, RSAD2, and OSAL are significantly up-regulated in COVID-19 patients and are involved in immune regulation (41). The infected macrophages of COVID-19 patients release large amounts of interferon into the blood, which activates mitochondrial IFI27 expression and disrupts the energy metabolism of immune cells (42). Genes such as IFI27 that exert antiviral effects and neutrophil activation are downregulated during treatment in COVID-19 patients, which is consistent with the dynamically enhanced inflammatory response of COVID-19 patients (43). In patients with influenza virus infection, IFFI44L, IFI6, and RSAD2 were key antiviral factors against IAV infection in alveolar basal epithelial cells (44). As a novel immune biomarker, IFI27 can accurately distinguish between influenza and bacterial infections (45).

The critical role of the immune response in infectious diseases has received increasing attention. In this analysis, we found that the numbers of activated CD8 T cells, effector memory CD4 T cells, memory B cells, and central memory CD8 T cells were significantly reduced in influenza and COVID patients. This is consistent with the findings of Yu Bai, which indicate that the number of CD4 and CD8 T cells in the peripheral blood was significantly decreased in COVID-19 patients and that the severe damage during the late stage was immune-related rather than virus-related (6). This study also showed that lymphocytopenia is common in COVID-19 patients, indicating both immune cell depletion and impaired cellular immune function (6). The hematological parameters for COVID-19 are similar. Cao et al. also found that half of the influenza patients during the 2009 H1N1 pandemic had abnormal CD4:CD8 ratios (46). Furthermore, we also found that the expression of the five hub genes was associated with Eosinophils, Type 17 T helper, T follicular helper, Natural killer T, Natural killer, and Immature B cells. These results demonstrate the importance of immune cell infiltration for the pathogenesis and typing of influenza and COVID-19. Previous reports have suggested a close relationship between the central genes identified in this study and immunity (27, 45, 47–49). Hub genes may be the main cause of these changes and may be used as immunotherapeutic targets.

In this study, we aimed to identify the effects of COVID-19 and influenza viruses on the regulation of host gene expression using a computational systems biology approach. Moreover, we explored the immune cell infiltration in COVID-19 and influenza. We used it to identify molecular mechanisms associated with comorbidities interactions, which may also facilitate the discovery of new knowledge from published datasets. However, our study has some limitations. First, external validation was not run for the results. Second, regarding the specificity of the blood sample, ssGSEA is a relatively quantitative result, and we could not achieve the accuracy required by the algorithm. Third, since the datasets included different ethnicities, it was difficult to accurately determine whether the different ethnicities could influence the related gene expression because of the small sample size. Therefore, our results still need further verification through *in vivo* and *in vitro* experiments and clinical studies.

## 5 Conclusion

In summary, five HUB genes were identified between COVID-19 and influenza virus infection, which might be helpful in the diagnosis and treatment of COVID-19 and influenza. Our results suggest that the regulatory effects exerted by both the influenza virus and COVID virus on host gene expression may be responsible for their similarities and differences in clinical manifestations. We provide molecular insights into potential biomarkers and regulatory elements that may contribute to developing novel drugs that can be used to control the progression of COVID-19 and influenza. The differentially expressed genes, GO terms, and signaling pathways identified in this study can help us gain a deeper understanding of how genes and clinical manifestations are associated. This knowledge can also guide future mechanistic studies that seek to develop pathogen-specific interventions.

## Data availability statement

The original contributions presented in the study are included in the article/**Supplementary Material**. Further inquiries can be directed to the corresponding authors.

## Author contributions

ZS and LK: Writing-original draft, Data analysis, performing the experiment; QZ, JQ, and YH: Data analysis, Writing-review & editing; HG and ZP: Manuscript revised. All authors contributed to the article and approved the submitted version.

## Supplementary material

Supplementary Figure 1 mRNA Expression levels of the 5 hub genes in GSE157103, GSE111368, and GSE101702 datasets between patients with different severity. **(A)** IFI6 in the GSE157103 dataset. **(B)** IFI27 in the GSE157103 dataset. **(C)** IFI44L in the GSE157103 dataset. **(D)** OASL in the GSE157103 dataset. **(E)** RSAD2 in the GSE157103 dataset. **(F)** IFI6 in the GSE111368 dataset. **(G)** IFI27 in the GSE111368 dataset. **(H)** IFI44L in the GSE111368 dataset. **(I)** OASL in the GSE111368 dataset. **(J)** RSAD2 in the GSE111368 dataset. **(K)** IFI6 in the GSE101702 dataset. **(L)** IFI27 in the GSE101702 dataset. **(M)** IFI44L in the GSE101702 dataset. **(N)** OASL in the GSE101702 dataset. **(O)** RSAD2 in the GSE101702 dataset.
