# Supplementary figures and images for: The use of bioinformatics methods to identify the effects of SARS-CoV-2 and influenza viruses on the regulation of gene expression in patients
Source: Front Immunol. 2023 Feb 22;14:1098688. doi: 10.3389/fimmu.2023.1098688 (PMC9992716; doi:10.3389/fimmu.2023.1098688)

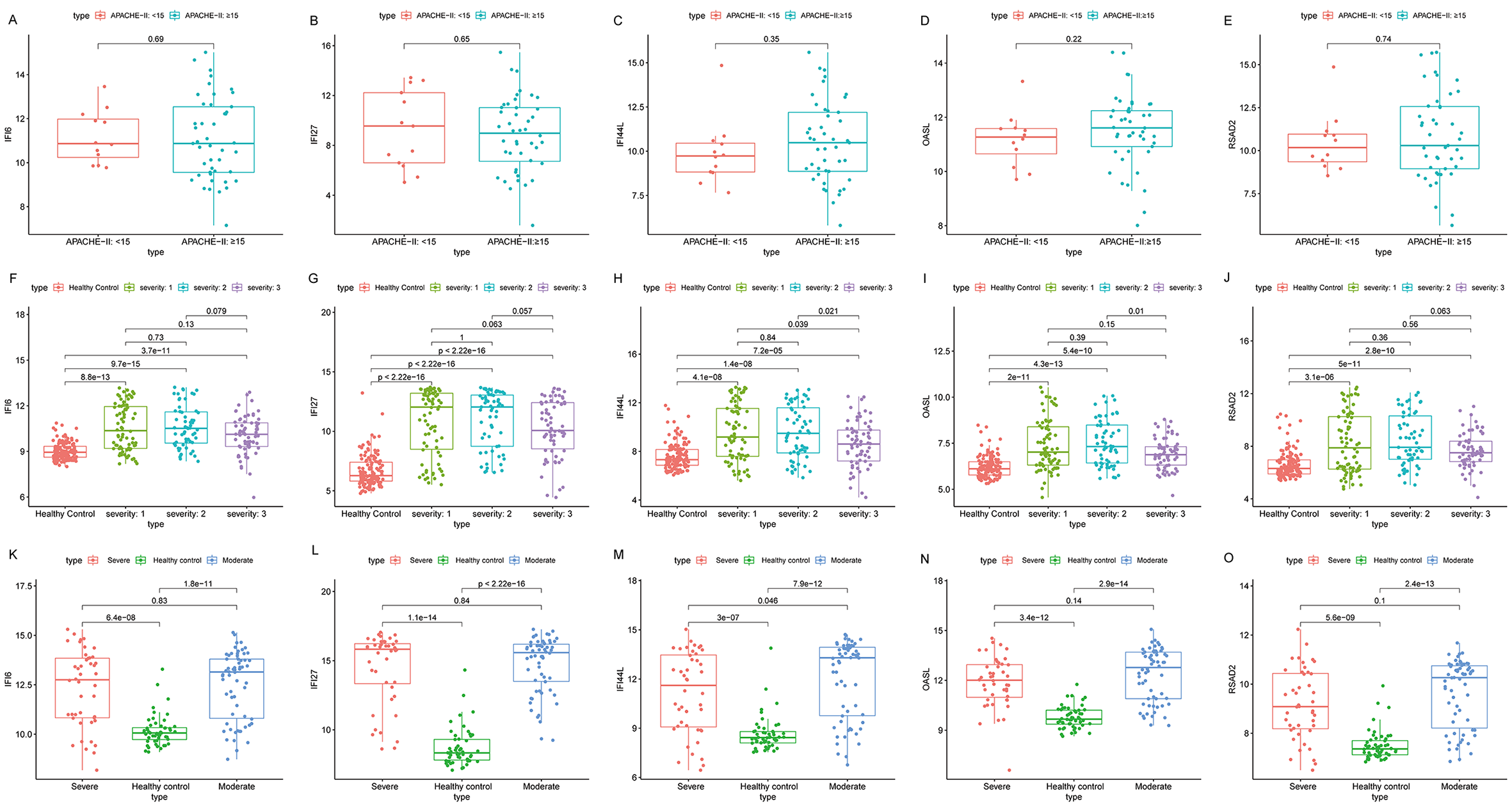

Supplement: Supplementary Figure 1 — mRNA Expression levels of the 5 hub genes in GSE157103, GSE111368, and GSE101702 datasets between patients with different severity. (A) IFI6 in the GSE157103 dataset. (B) IFI27 in the GSE157103 dataset. (C) IFI44L in the GSE157103 dataset. (D) OASL in the GSE157103 dataset. (E) RSAD2 in the GSE157103 dataset. (F) IFI6 in the GSE111368 dataset. (G) IFI27 in the GSE111368 dataset. (H) IFI44L in the GSE111368 dataset. (I) OASL in the GSE111368 dataset. (J) RSAD2 in the GSE111368 dataset. (K) IFI6 in the GSE101702 dataset. (L) IFI27 in the GSE101702 dataset. (M) IFI44L in the GSE101702 dataset. (N) OASL in the GSE101702 dataset. (O) RSAD2 in the GSE101702 dataset. [file Image_1.tif]
